# HIV-1 genetic diversity and demographic characteristics in Bulgaria

**DOI:** 10.1371/journal.pone.0217063

**Published:** 2019-05-28

**Authors:** Erik Billings, Richard A. Heipertz, Tonka Varleva, Eric Sanders-Buell, Anne Marie O'Sullivan, Meera Bose, Shana Howell, Gustavo H. Kijak, Hristo Taskov, Ivailo Elenkov, Marina Nenova, Nedialka Popivanova, Aimee Bolen Valenzuela, Otha Myles, Christian T. Bautista, Merlin L. Robb, Nelson L. Michael, Jerome H. Kim, Paul T. Scott, Sodsai Tovanabutra, Julie A. Ake

**Affiliations:** 1 United States Military HIV Research Program, Silver Spring, MD, United States of America; 2 The Henry M. Jackson Foundation for the Advancement of Military Medicine, Bethesda, MD, United States of America; 3 The Walter Reed Army Institute of Research, Silver Spring, MD, United States of America; 4 Ministry of Health, Department for Prevention and Control of AIDS, TB, and STIs, Sofia, Bulgaria; 5 National Center for Infectious and Parasitic Diseases, Sofia, Bulgaria; 6 Hospital of Infectious Diseases Prof. Iv. Kirov, Department for Patients with Acquired Immune Deficiencies, Sofia, Bulgaria; 7 Medical University Hospital, St. Marina, Infectious Diseases Clinic, Varna, Bulgaria; 8 Medical University Hospital St. Georg, Infectious Diseases Clinic, Plovdiv, Bulgaria; Hebei Provincial Center for Disease Control and Prevention, CHINA

## Abstract

HIV-1 strain diversity in Bulgaria is extensive and includes contributions from nearly all major subtypes and the Circulating Recombinant Forms (CRF): 01_AE, 02_AG, and 05_DF. Prior to this study, HIV-1 sequence information from Bulgaria has been based solely on the *pro-RT* gene, which represent less than 15% of the viral genome. To further characterize HIV-1 in Bulgaria, assess participant risk behaviors, and strengthen knowledge of circulating strains in the region, the study “Genetic Subtypes of HIV-1 in Bulgaria (RV240)” was conducted. This study employed the real time-PCR based Multi-region Hybridization Assay (MHA) B/non-B and HIV-1 sequencing to survey 215 of the approximately 1100 known HIV-1 infected Bulgarian adults (2008–2009) and determine if they were infected with subtype B HIV-1. The results indicated a subtype B prevalence of 40% and demonstrate the application of the MHA B/non-B in an area containing broad HIV-1 strain diversity. Within the assessed risk behaviors, the proportion of subtype B infection was greatest in men who have sex with men and lowest among those with drug use risk factors. During this study, 15 near full-length genomes and 22 envelope sequences were isolated from study participants. Phylogenetic analysis shows the presence of subtypes A1, B, C, F1, and G, CRF01_AE, CRF02_AG, CRF05_DF, and one unique recombinant form (URF). These sequences also show the presence of two strain groups containing participants with similar risk factors. Previous studies in African and Asian cohorts have shown that co-circulation of multiple subtypes can lead to viral recombination within super-infected individuals and the emergence of new URFs. The low prevalence of URFs in the presence of high subtype diversity in this study, may be the result of successful infection prevention and control programs. Continued epidemiological monitoring and support of infection prevention programs will help maintain control of the HIV-1 epidemic in Bulgaria.

## Introduction

The HIV-1 epidemic in Bulgaria is notable for its high level of strain diversity and relatively low amount of new and existing infections. Recent studies using *pro-RT* sequences have reported the presence of subtypes A1, B, C, F1, and H as well as CRFs: 01_AE, 02_AG, 05_DF, and URFs in Bulgaria [1, 2]. Such broad diversity is perhaps unexpected given new HIV-1 diagnoses varying between 171 in 2009 to 247 in 2014 [3] and an HIV-1 prevalence of 0.015% (1109 known infections) in 2009 [4], all of which are well below the rates of Western & Central Europe (estimated new infections: 29,000 and prevalence: 0.2% in 2012) or North America (estimated new infections: 48,000 and prevalence: 0.5% in 2012) [5]. However, while the rates of infection appear low, Bulgaria’s geographic position along major transcontinental shipping routes and its role as a point of origin for human trafficking [6] makes characterizing and monitoring the high level of HIV-1 strain diversity within Bulgaria an important concern. Owing to its location in the eastern part of the Balkan Peninsula, Bulgaria is bordered on three sides by countries that contain very different collections of HIV-1 strains.

To the north of Bulgaria, Romania contends with an HIV-1 epidemic that over the past decade has been found to contain largely subtype F1 strains (>70%) followed by subtype B (6–14%) and subtype C (4%) [7–9]. To the west, Serbia has an epidemic that is predominantly subtype B (>90%) with a small number of infections caused by subtypes A1, F1, CRF01_AE, and CRF02_AG [9, 10]. The other western neighbor, the former Yugoslav Republic of Macedonia, is known to have active HIV-1 and AIDS cases [3, 11], however reported cases are few in number (less than 200 as of 2014) and have yet to be molecularly characterized [12] or sequenced and published as of August, 2018. Bulgaria’s southwestern neighbor, Greece, has an HIV-1 epidemic that is mostly non-subtype B [13], with subtype B represented at less than 46% and the balance containing subtype A1 (41%) and a mix of subtypes C, F1, G, and recombinants including CRFs: 01_AE, 02_AG, 04_cpx, and 11_cpx [9]. To the southeast, Turkey has an epidemic that contains a similar proportion of subtype B (45%) as Greece, but differs in its remaining constituents, which are mostly B/F recombinants (22%), subtypes A1 (12%) and F1 (7%) along with smaller amounts of CRF02/B recombinants and subtypes C, G [9, 14]. Within those settings, this study performed the systematic and detailed characterization of HIV-1 subtype diversity of circulating strains in Bulgaria.

The RV240 Genetic Subtypes of HIV-1 in Bulgaria 2008–2009 study, in a collaboration between the Bulgarian Ministry of Health, the Bulgarian National Center for Infectious and Parasitic Diseases, the Naval Medical Research Unit No. 3, and the U.S. Military HIV Research Program performed characterization of circulating HIV-1 strain diversity by implementing a high-throughput real-time PCR based assay that distinguishes subtype B from non-subtype B strains. The Multi-region Hybridization Assay (MHA) B/non-B [15] uses subtype B specific probes to identify subtype B strains through probe hybridization to six regions across the HIV-1 genome. Additionally, since current sequence knowledge of HIV-1 in Bulgaria is limited to the protease and RT genes, complete envelope gene and near full-length HIV-1 genome sequencing was conducted within subsets of study participants for phylogenetic analysis and MHA assay validation. Herein, we describe high-throughput measurements of strain diversity using the MHA B/non-B assay as well as the molecular epidemiology of *env* gene and full-length genomes of circulating strains in Bulgaria. The combined results indicate a diverse collection of subtype and recombinant strains and provide a previously unavailable sequence and molecular epidemiology status of HIV-1 in Bulgaria.

## Materials and methods

### Study participants

Between December 2008 and January 2010, the RV240 Genetic Subtypes of HIV-1 in Bulgaria 2008–2009 study (human use protocol #W1424 [RV240]) enrolled 377 HIV-1 infected volunteers from HIV-1 anti-retroviral treatment clinical sites in the cities of Sofia (n = 281), Varna (n = 54), Plovdiv (n = 33), and others (n = 9). The study was reviewed and approved by the institutional review boards of the Walter Reed Army Institute of Research and the Naval Medical Research Unit No.3 as well as the ethical review committees of the National Center for Infectious and Parasitic Diseases, Sofia Infectious Disease Hospital, Saint Georg Medical University Hospital, and Saint Marina Medical University. Participant consent was obtained prior to enrollment. During enrollment, participants were surveyed for risk factors such as commercial sex work (CSW) received or paid, drug use (intravenous or non-intravenous), men having sex with men (MSM), sex while traveling (casual or with travel companion), and transfusion. Peripheral blood mononuclear cell (PBMC) fractions from 343 participants were used to perform PCR amplification of HIV-1. Participant viral loads were not collected. Bonferroni-corrected one-way ANOVA comparisons of participants’ demographic attributes and verification of distribution type was performed using GraphPad Prism version 7.0 (GraphPad Software, La Jolla, CA).

### Development of MHA B/non-B assay

**Fig 1:**
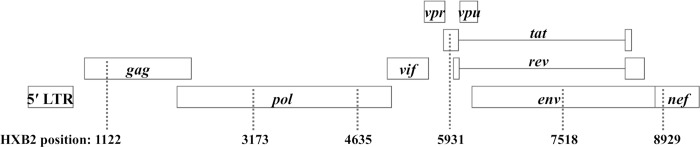
MHA B/Non-B probe locations. HXB2 referenced positions of the six probe locations in the HIV-1 genome.

Development of the MHA B/non-B assay required the design and testing of universal primers and subtype B specific fluorescent probes. Using available sequences (hiv.lanl.gov), six locations within the HIV-1 genome were identified as containing enough similarity to allow amplification of all HIV-1 subtypes and main CRFs, while simultaneously possessing enough heterogeneity to allow the probes to distinguish between subtype B and non-B strains. The amplified regions varied in length from 216 to 379 nucleotides and were located in *gag* (MA), *pol* (RT and INT), *tat*, *env* (C4), and *nef*, Fig 1. The probes were designed to react with subtype B strains. Targeted region amplification was monitored by incorporation of SYBR Green within the control reactions for each combination of sample and primers. Viral strains classified as subtype B displayed positive probe reactivity with at least 4 of the targeted regions.

### MHA B/non-B template, PCR, and probes

DNA was extracted from 2.5 to 5 million cells of PBMC using the MagNA Pure robotic nucleic acid extraction procedure (Roche Diagnostics, Indianapolis, IN), according to the manufacturer’s instructions. The extracted DNA was used in first-round PCR containing: GeneAmp 10x PCR Gold Buffer (Applied Biosystems), 200 μM each dNTP, 1.5 mM MgCl_2_, 400 nM of each outer primer, 1.25 U of AmpliTaq Gold DNA Polymerase (Applied Biosystems), and 2 μL of the extracted DNA in a final volume of 50 μL per reaction. The thermocycler routine was: hold at 95°C for 10 min, then 45 cycles of 94°C 15 s, 60°C 1 min, and 72°C 10 min, and a final extension at 72°C for 10 min in a PerkinElmer 9600 thermocycler (PerkinElmer Inc., Waltham, MA). An aliquot of the first round PCR product was used as the template for second-round PCR, which was conducted in a TaqMan real-time format, with universal primers and subtype B specific fluorescent probes. The sequences and positions of the primers and probes are listed in the S1 Table. All probes were 5’-end labeled with FAM and 3’-end labeled with BHQ1. The second-round real-time PCR mix contained TaqMan 2x Universal PCR Master Mix (Applied Biosystems Inc.), 400 nM of each inner primer, 250 nM of fluorescent probe, and 2.5 μL of the first-round PCR product in a final volume of 12.5 μL. Real-time PCR amplification was performed in a 384-well ABI PRISM 7900HT Sequence Detection System (Applied Biosystems) using the conditions: hold at 95°C for 10 min, 40 cycles of 95°C 15 s to 55°C 1 min, and 60°C 1 min.

### MHA B/non-B primer and probe testing

Details of the initial testing of primers and probes for each region have been described previously [16–19]. Briefly, primers were tested with a panel of full-length HIV-1 DNA generated from previously characterized samples. Probes were tested for functionality with DNA fragments that have a 5–10 nucleotide overhang on each end with respect to the probe length. For each probe and target pair, a reaction mix containing 2x TaqMan SYBR Green PCR Master Mix (Applied Biosystems Inc., Foster City, CA), 1 μM probe, 1 μM target, and dH_2_O was prepared in duplicate. Dissociation curves were obtained with a 384-well ABI PRISM 7900HT Sequence Detection System (Applied Biosystems Inc., Foster City, CA) using the following conditions: 95°C for 15 s, 30°C for 15 s, and 95°C for 15 s at a 10% ramp rate. The melting temperature of the duplexes was estimated as the maximum of the peak in the–dRn/dT vs. temperature graph.

### MHA data processing

During the reaction, fluorescence intensity was monitored and analyzed using the SDS v2.1 software (Applied Biosystems Inc.). Positive results were defined as having a threshold cycle lower than 35 cycles and having displayed an exponential increase in the normalized fluorescence intensity for five or more consecutive cycles. The PCR positive control for each sample was conducted in parallel with the other reactions to assess whether negative real-time PCR results were due to a lack of template amplification or probe hybridization. Control reactions contained 2x SYBR Green PCR Master Mix (Applied Biosystems Inc.) and similar amounts of inner primers and template as described above. Within the control reactions, amplicon production was confirmed using melting curves (95°C 15 s, 30°C 15 s, and 95°C 15 s at 10% ramp rate) to distinguish the expected PCR products from primer-dimers of lower thermal stability.

### Sequencing

Fifteen of the subtyped samples were subjected to near full-length sequencing and 22 other samples were subjected to *env* sequencing. PBMC DNA provided the template to generate full-length and *env* sequences using amplification and sequencing methods as previously described [20]. Sequence data were assembled and manually edited using Sequencher 5.0 (Gene Codes Corporation, USA).

### Phylogenetic analysis

The initial HIV-1 genotype was assigned for each sequence in this study using the HIV-1 Genotyping Tool at the National Center for Biotechnology Information (NCBI) [21]. A multiple sequence alignment of the sequences from this cohort including subtype reference sequences was constructed with HIVAlign [22] and refined using MEGA version 5 [23]. Neighbor-Joining trees were constructed and bootstrap values (maximum likelihood, 100 replicates) supporting relevant branches were calculated with DIVEIN [24] using the estimated GTR+I+G model. Phylogenetic trees generated from the analysis were visualized using FigTree version 1.4.2 (http://tree.bio.ed.ac.uk) and analyzed for genotype assignment. Informative site analysis, visual inspection, bootstrap analysis of subgenomic trees, and the jumping profile Hidden Markov Model analytical tool [25] were used to precisely map breakpoints within the final genome structures of inter-subtype recombinants [26–28].

## Results

Validation of the MHA probes and primer sequences was conducted using a subtype reference panel of 35 full-length HIV-1 genomes representing subtypes A (n = 4), B (n = 7), C (n = 8), D (n = 6), G (n = 2), CRF01_AE (n = 5), CRF02_AG (n = 2), and one A1G recombinant as listed in the S2 Table. Scoring of the validation results was assessed per amplified region and included amplification success, probe reactivity for the known subtype B genomes, and probe specificity which quantified the number of correct probe reactions in the validation panel. Across the six genomic regions: amplification success ranged from 83–100%, probe sensitivity ranged from 80–100%, and probe specificity ranged from 88–100% as detailed in Table 1. MHA B/non-B performance within the study cohort was confirmed by testing assay performance on a selection of 15 full-length HIV-1 genomes from participant samples which had been subjected to full-length HIV-1 sequencing. Among those samples, amplification success ranged from 87–93%, probe sensitivity ranged from 80–100%, and probe specificity ranged from 85–100%, Table 2.

**Table 1:** MHA B/non-B validation subtype reference panel results.

	*gag*	*pol*	*int*	*tat*	*env*	*nef*
Amplification success	97%	97%	100%	83%	94%	94%
Probe Sensitivity	100%	86%	86%	100%	80%	100%
Probe Specificity	91%	97%	94%	100%	88%	94%

**Table 2:** MHA B/non-B participant reference panel results.

	*gag*	*pol*	*int*	*tat*	*env*	*nef*
Amplification success	93%	87%	93%	87%	93%	87%
Probe Sensitivity	100%	100%	100%	100%	100%	80%
Probe Specificity	100%	100%	93%	100%	93%	85%

### HIV-1 subtype distribution and phylogeny in Bulgaria

**Fig 2:**
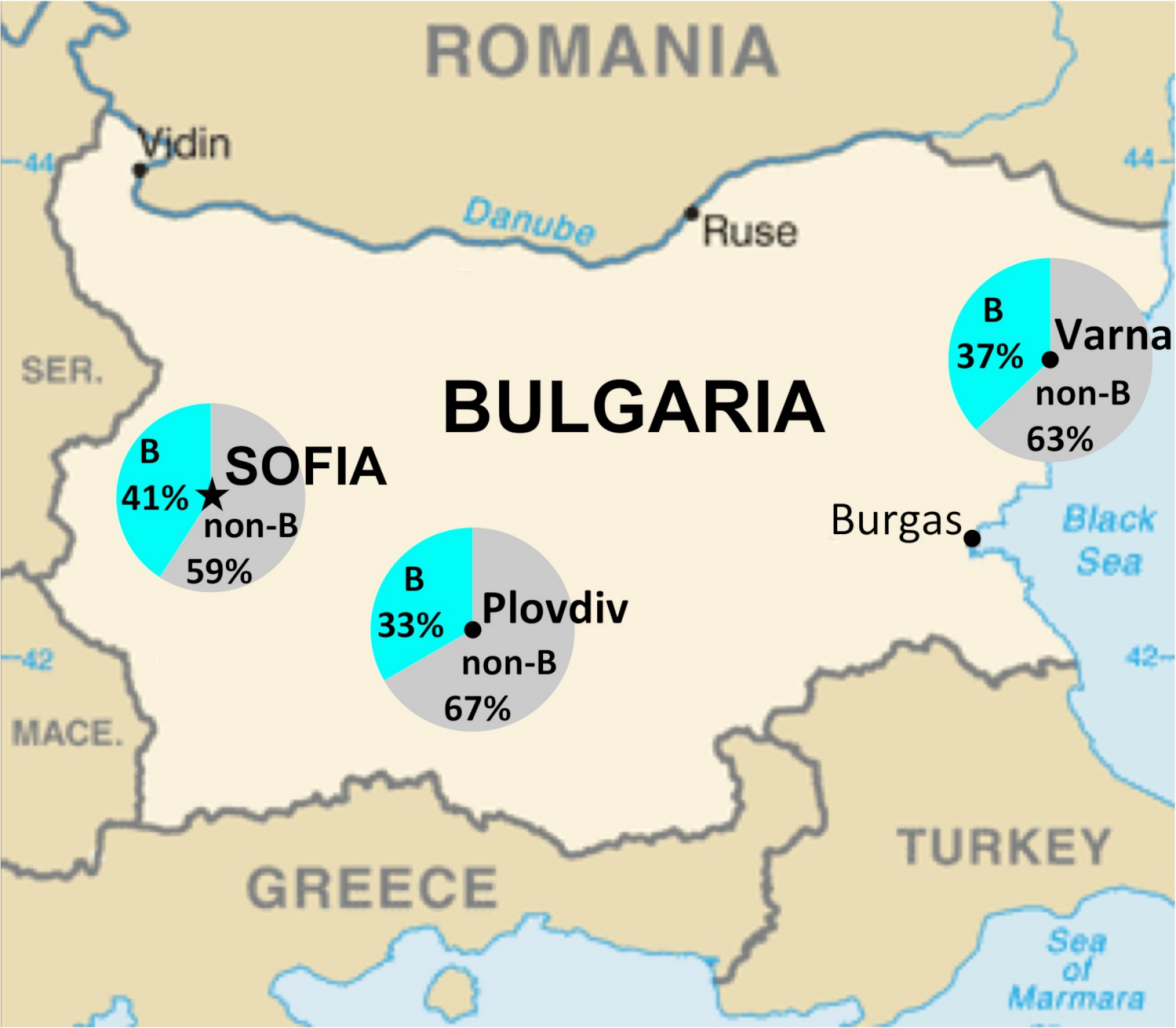
HIV-1 subtype distribution across three study sites in Bulgaria. Proportion of subtype B and non-B infections at each of the three study sites.

**Fig 3:**
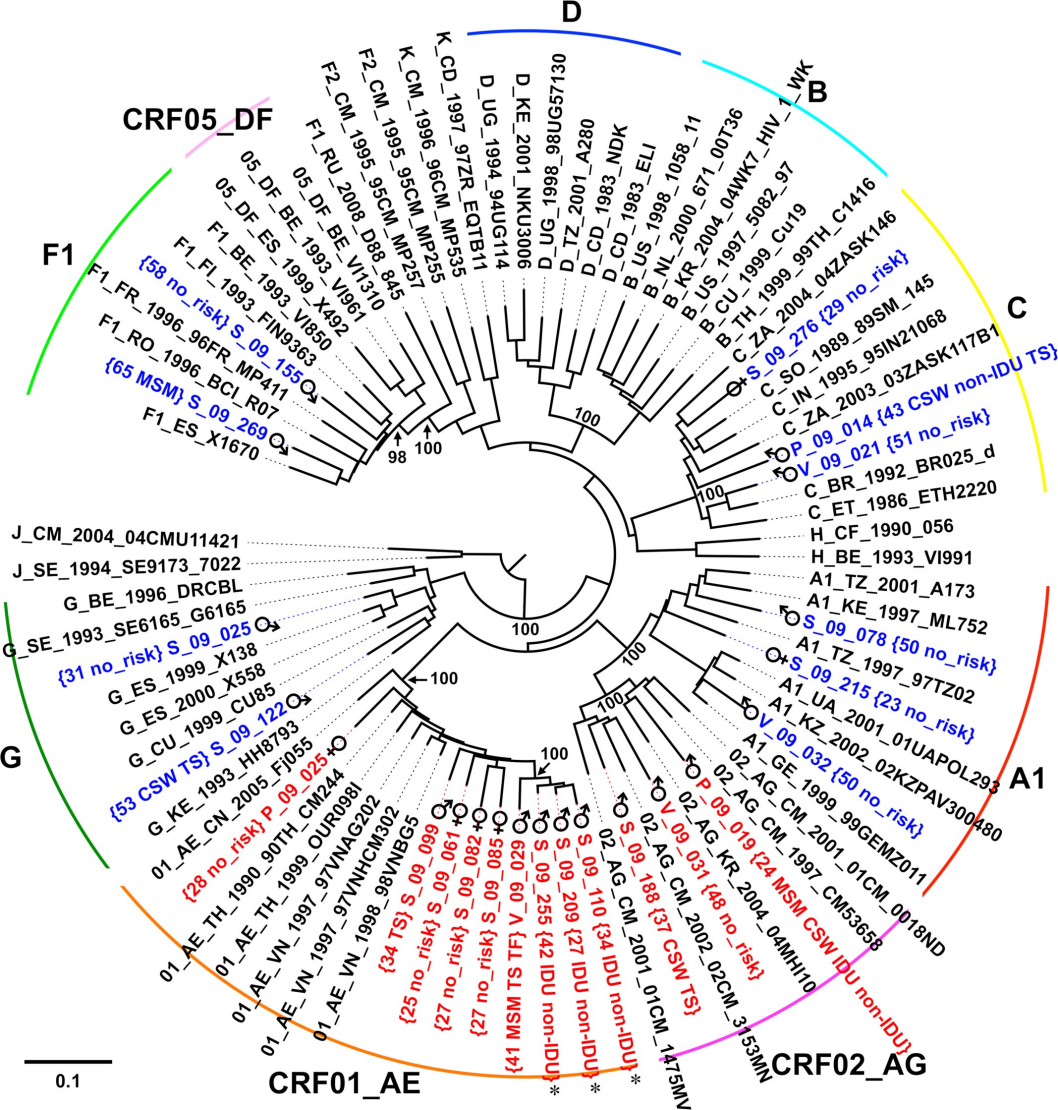
Phylogenetic tree of HIV-1 *env* sequences from Bulgaria. Neighbor-joining tree of *env* gene sequences denoted with group subtypes, participant age at enrollment, gender, and self-identified risk factors. Study participant strains from pure subtypes are shown in blue and recombinant strains are shown in red. The trio of males with intra-group genetic distances less than 3% are marked with *. Bootstrap values at relevant nodes are shown. The scale bar indicates a genetic distance of 10%.

**Fig 4:**
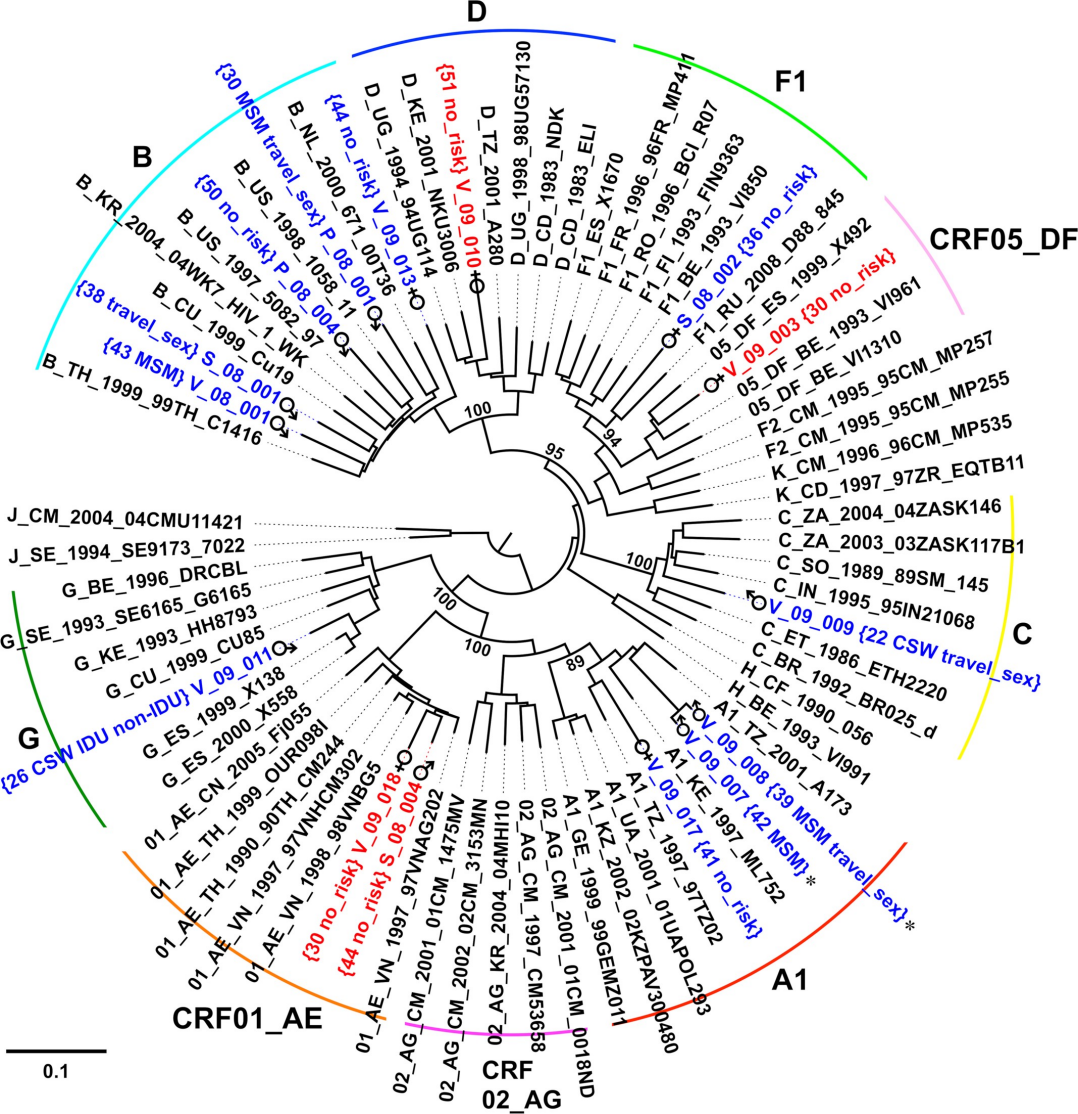
Phylogenetic tree of full-length HIV-1 sequences from Bulgaria. Neighbor-joining tree of full-length sequences denoted with group subtypes, participant age at enrollment, gender, and self-identified risk factors. Study participant strains from pure subtypes are shown in blue and recombinant strains are shown in red. The closely related pair of strains with an *env* genetic distance of 2.5% are marked with *. Bootstrap values at relevant nodes are shown. The scale bar indicates a genetic distance of 10%.

From the 343 participant PBMC fractions, only 276 contained sufficient viral DNA to produce genomic template for characterization. Of those, 61 (22%) did not amplify at least four of the six targeted regions in the HIV-1 genome and were therefore classified as non-typeable. The results from the remaining 215 typeable samples (166 Sofia, 31 Varna, 18 Plovdiv) revealed an overall subtype B proportion of 39%, which ranged from 33% to 41% between the three study sites (Fig 2) and was nonuniformly distributed between the male and female participants (Table 3). During the MHA’s development, 15 full-length HIV-1 genome sequences were produced using samples from early enrollees. Once the MHA was completed, 22 additional non-subtype B samples were selected for sequencing of the *env* gene to obtain sequence information that would be useful for future vaccine development in Bulgaria and the surrounding region. Phylogenetic analysis of the *env* sequences revealed an extensive diversity of strains, which include subtypes A1, C, F1, G, CRF01_AE, and CRF02_AG. Within the *env* dataset, a group of CRF01_AE strains was observed with intra-group genetic distances of less than 3% from a trio of males with self-identified intravenous drug use (IDU) and non-IDU risk behaviors, Fig 3. Among the full-length sequences, phylogenetic analysis revealed the presence of a closely related pair (*env* genetic distance: 2.5%) of subtype A1 strains from participants with MSM risk behaviors. Similar to the *env* analysis, multiple subtypes were observed, including A1, B, C, F1, G, CRF01_AE, CRF05_DF, and an A1/C/D unique recombinant form (URF), Fig 4. Genomic breakpoint analysis confirmed the presence of CRF05_DF and determined the structure of the A1/C/D URF strain, S1 Fig.

**Table 3:** Number of typeable participants with detected subtypes, by location and gender.

	Plovdiv		Varna		Sofia	
	subtype B	non-B	subtype B	non-B	subtype B	non-B
Male	5	5	6	13	60	61
Female	1	7	4	8	9	36

### Demographic attributes

In this study, male participants outnumbered females by more than 2-fold. The ratio of male-to-female participants from the main study sites were: Plovdiv 20/13, Varna 35/19, and Sofia 194/87; the nine participants from other cities were male. Within the subtyped samples, 150 were from male participants (median age: 39 years, interquartile range *[IQR]*: 32–47 years) and 65 were from female participants (median age: 33 years, *IQR*: 28–40 years). Among those groups, 79% of the males reported having one or more of the listed risk behaviors whereas only 23% of the females acknowledged a risk behavior. In general, most participants who reported any risk factor, typically selected more than one. The queried risk factors were: commercial sex work (CSW) received or paid, intravenous drug use (IDU) or non-IDU, men having sex with men (MSM), sex while travelling (casual or with a travel companion), and transfusion. In this report, the CSW and sex while travelling risk factors are grouped respectively into CSW and travel sex.

The number of typeable participants self-indicating one or more risk factors, their gender, and the detected infection subtype are shown in Table 4. The low number of risk responses from the female participants prevents dissection of female risk behaviors in relation to the infecting subtypes. However, among all subtyped females 22% are infected with subtype B HIV-1 strains, whereas 47% of the males are infected with B strains. Dissection of the male self-reported risks by subtype shows IDUs (13%) and non-IDUs (35%) having the lowest percentage of subtype B infections and MSM having the highest percentage of subtype B infections (70%). Within the transfusion, CSW, and travel sex risk categories (males only) the percentage of subtype B infections detected were 40%, 42%, and 56%, respectively. Further dissection of the male risks, Table 5, shows significant (*p* values < 0.02) age differences of greater than 10 years between the drug use category and the CSW, travel sex, and transfusion risk categories. Conversely, within each behavior category, small differences in age (less than 4 years) were observed between subtype B or non-B infected male participants. For both genders, the proportion of subtype B infections above and below the median ages (males: 39 years, females: 33 years) were 49% and 45% for males and 21% and 22% for females, respectively, with no statistically significant differences.

**Table 4:** Number of typeable participants by gender, subtype, and self-reported risk behavior.

**Risk Factor**	**Male**		**Female**	
	B	Non-B	B	Non-B
CSW	24 (42%)	33 (58%)	0	4 (100%)
IDU	2 (13%)	14 (87%)	1 (17%)	5 (83%)
Non-IDU	9 (35%)	17 (65%)	2 (29%)	5 (71%)
MSM	43 (70%)	18 (30%)	-	-
Travel sex	41 (56%)	32 (44%)	1 (50%)	1 (50%)
Transfusion	6 (40%)	9 (60%)	3 (50%)	3 (50%)
Any risk	61 (52%)	57 (48%)	5 (33%)	10 (67%)
No risk	10 (31%)	22 (69%)	9 (18%)	41 (82%)
All participants	71 (47%)	79 (53%)	14 (21%)	51 (78%)

Proportions show subtype distribution within each gender-risk category.

**Table 5:** Mean age (years) of typeable participants by gender, subtype, and self-reported risk behavior.

**Risk Factor**	**Male**		**Female**	
	B	Non-B	B	Non-B
CSW	39.7	39.5	-	29.5
IDU	30.5	28.8	31	27.2
Non-IDU	29	29.2	32.5	26
MSM	36.9	37.3	-	-
Travel sex	39.4	40.9	27	30
Transfusion	41.2	44.6	31.7	33.7
Any risk	39.4	38.9	32.1	30.1
No risk	45.8	42.4	36.2	37.8
All participants	40.3	39.8	34.2	36.3

## Discussion

This study describes the characterization of HIV-1 genetic diversity in Bulgaria using a combination of the high-throughput real-time PCR based Multi-region Hybridization Assay (MHA) B/non-B and *env*/full-length genome sequencing. Although 343 participant samples were received and analyzed, 67 did not produce genomic HIV-1 template for subsequent assay steps, this was likely due to the use of HIV-1 therapy clinics as the primary recruitment sites and the enrollment of long-term viral suppressed participants. In addition, another 61 samples were classified as non-typeable since they did not amplify at least four of the six targeted regions. This was potentially due to low viral loads, but could also result from the presence of recombinants containing genomic contributions from less common CRFs that were not included during the design of the universal primers. For the remaining 215 samples, the MHA B/non-B assay demonstrated target amplification, probe sensitivity and probe specificity across six regions of the HIV-1 genome (*gag*, *RT*, *int*, *tat*, *env* and *nef*). This strategy provides for subtype determination based on multiple distant regions of the HIV-1 genome instead of relying on partial sequencing or amplification and analysis of a single subgenomic region. MHA B/non-B analysis revealed an overall subtype B prevalence of 39%, which is less than the proportion of *prot-RT* based subtype B strains (50%) currently available in the HIV-1 database (hiv.lanl.gov) for Bulgaria during the study enrollment period. The difference between these proportions is likely due to the limited genomic information obtained when analyzing a single region such as *prot-RT*.

When stratified by gender, subtype B infections were detected in 47% of the subtyped males and 21% of the subtyped females. Further stratification of the males by self-reported risk factors disclosed a nonuniform distribution wherein males reporting activities such as MSM or travel sex had the highest percentage of subtype B infections (70% and 56%, respectively), while males reporting drug use activities (IDU or non-IDU) have the lowest percentage of subtype B infections (13% and 35%, respectively). The high percentage of subtype B strains among the MSM risk group and low percentage of subtype B strains among the females are both consistent with previously identified trends in European countries [29, 30]. The lower percentage of subtype B infections in the male IDU risk group (13%) sampled in this study is intermediate to that seen in previous reports of strain diversity in IDU from general European (61.4%) [30] and Bulgarian (8.5%) [2] cohorts. Overall, these results are consistent with the finding that MSM have larger proportions of subtype B infections than IDU or heterosexual risk groups in Europe.

When examined in terms of participant age, the proportion of subtypes were equivalent above and below the median ages regardless of gender. Combined with the low number of self-identified risk behaviors among the females, these results support the premise that males are the primary link between these risk behaviors and their female partners [4]. In addition, within the risk categories there were no significant differences in the mean ages of participants infected with subtype B or non-B strains. This suggests a consistent and established HIV-1 subtype distribution within the behavioral groups queried by this study. In contrast, there were differences in the ages of males between the risk categories. Significant differences in male participant ages were observed between the drug use category (mean ages of 29 and 29.1 years for IDU and non-IDU, respectively) vs the CSW (*p* = .017), travel sex (*p* = .002), and transfusion (*p* = .013) categories with mean ages of 36, 40, and 43 years, respectively. Although slightly older, the relative youth of participants in this study’s drug use category is similar to a recent report, which found a higher proportion of youths among the people who inject drugs demographic with a mean age of 26.4 years for both genders [2].

Analysis of the *env* and full-length sequences identified two small groups of participants with similar risk factors: a triplet of drug users infected with CRF01_AE (Fig 3) and a pair MSM infected with subtype A1, Fig 4. Although these were small sample subsets, the presence of closely related strain groups (*env* genetic distance < 3%) suggests these transmission events occurred within Bulgaria and are not simply the result of infection during foreign travel. Given the high diversity of HIV-1 strains in Bulgaria, the number of unique recombinant forms (URF) observed in this study was surprisingly low. Since previous analyses were limited to *pro-RT* sequences, it was expected that multiple URFs would be observed once longer *env* or full-length sequences were obtained. Other countries with comparable diversity [31, 32], displayed high proportions of URF (approaching half of the sequences collected), which should have been apparent within the 37 sequences collected here. Aside from potential sampling bias, the most likely explanation for the low number of URFs is the low prevalence of HIV-1 in Bulgaria, which would minimize the opportunity for super-infection and recombinant generation. In either instance, these sequences are a valuable reference for future epidemiology studies and vaccine development.

In conclusion, this study sampled and subtyped HIV-1 strains from 215 of the approximately 1100 known Bulgarian HIV-1 infections. The MHA B/non-B assay provides a high-throughput and high-resolution means for subtyping HIV-1 infections. Compared to analysis of a single genomic region, the resolution generated by assaying multiple regions of the HIV-1 genome, provides greater confidence in subtype assignments in countries where multiple subtypes or recombinants are circulating. However, any subtyping assay that makes sub-genomic measurements has the potential to miss and under-report recombination events that occur outside of the observed regions (a limitation only overcome by resource intensive full-length sequencing). The high diversity of HIV-1 strains sequenced during this study indicate multiple introductions of HIV-1 into the Bulgarian populace and are representative of strains found among its neighboring countries as well as distant continents. Fortunately, new infection rates in Bulgaria are low compared to Europe in general. However, if new infection rates increase to levels where super-infections become common, then the established HIV-1 subtype diversity can be expected to contribute to the production of new URFs. In the context of vaccine development and monitoring of the epidemic, increased strain diversity complicates the immunogen or reagent selection process. Given the high level of strain diversity already present in the Bulgarian epidemic, continued monitoring for changes in subtype distribution and continued support for testing, treatment, and prevention programs will be necessary to maintain control of HIV-1 in Bulgaria.

## Sequence data

The 37 HIV-1 sequences produced during this study have been submitted to GenBank and are available under accession numbers MH746230-MH746267.

## Supporting information

S1 Fig Genomic structures of observed URF_A1/C/D and CRF05_DF strains. Genomic subtype breakpoints of this study’s A1/C/D URF and CRF05_DF strains relative to the position of HIV-1 genes.
(TIF)

S1 Table Primer and probe sequences. (DOCX)

S2 Table MHA B/non-B validation panel. (DOCX)
