# Supplementary figures and images for: HIV-1 genetic diversity and demographic characteristics in Bulgaria
Source: PLoS One. 2019 May 28;14(5):e0217063. doi: 10.1371/journal.pone.0217063 (PMC6538145; doi:10.1371/journal.pone.0217063)

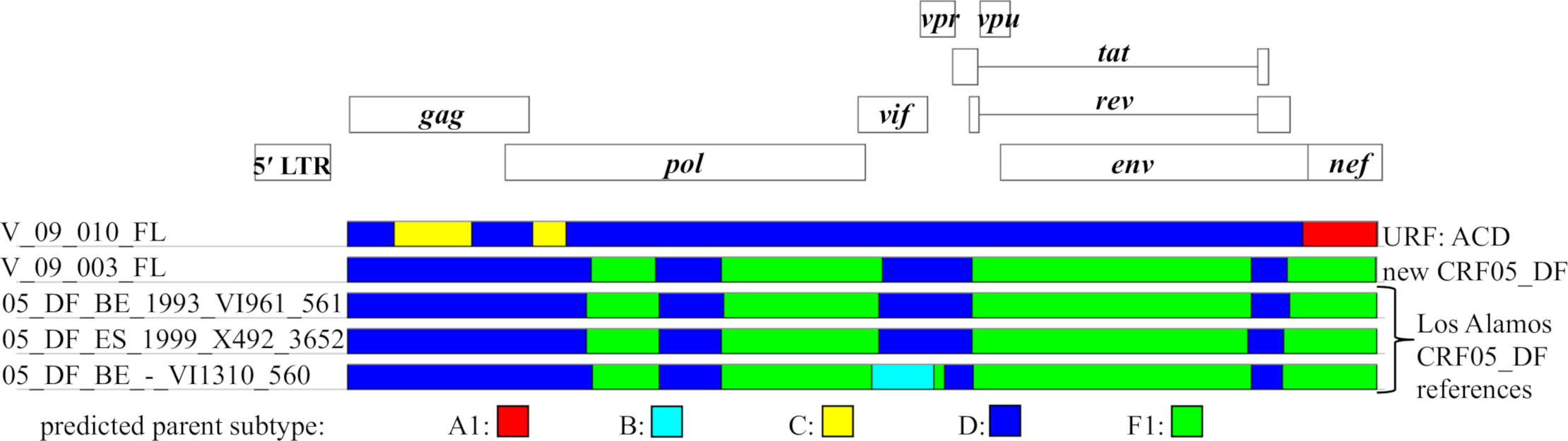

Supplement: S1 Fig — Genomic subtype breakpoints of this study’s A1/C/D URF and CRF05_DF strains relative to the position of HIV-1 genes. (TIF) [file pone.0217063.s001.tif]
