# Supplementary material for: HIV-1 genetic diversity and demographic characteristics in Bulgaria
Source: PLoS One. 2019 May 28;14(5):e0217063. doi: 10.1371/journal.pone.0217063 (PMC6538145; doi:10.1371/journal.pone.0217063)
Supplement: S1 Table — (DOCX) [file pone.0217063.s002.docx]

**S1 Table. Primer and Probe Sequences**

| Probe Position  (HXB2 number) | Primer/Probe | Sequence (5’ – 3’) |
| --- | --- | --- |
| *gag* | gag 1.0_5'outer5 | AAACATYTAGTATGGGCAAGCA |
| (1108 – 1136) | gag 1.0_3'outer1 | ACTTTYACCCATGCATTYAAAGT |
|  | gag 1.0_5'inner2 | ATAGAGGTAAAAGACACCAAGGAAGC |
|  | gag1.0_3'inner1 | TGCACTATAGGRTAATTTTGRCTG |
|  | B_PR2_gag1.0v2 | FAM-TGTGCYTTTTTCTTACTTTTGTTTTGCTC-BHQ1 |
| *pol* | pol_1.0_5'_outer2 | CCARAGTAGCATGACAAAAATCTTAGA |
| (3159 – 3187) | pol_1.0_3'_outer2 | AGGATGGAGTTCATAHCCCATC |
|  | pol_1.0_5'_inner2 | TATGTAGGATCTGAYTTAGARATAGG |
|  | pol_1.0_3'_inner2 | CATCCAAAGRAATGGRGGTTC |
|  | B_PR2_pol_1.2 | FAM-CCCCACYTCAACAGATGTTSTCTCAGTTC-BHQ1 |
| *int* | int _1.0_5'_Outer | AAAATTAGCAGGAAGATGGCCAG |
| (4622 – 4645) | int _1.0_3'_Outer | CTGCTGTCTTAAGRTGYTCAGC |
|  | int _1.0_5'_Inner | GTTAARGCMGCCTGTTGGTGG |
|  | int_1.0_3'_Inner | CTACTCCYTGACTTTGGGGATTGTA |
|  | B_PR2_INT_1.0 | FAM-AATTCCTGCTTGATCCCCGCCCAC-BHQ1 |
| *tat* | tat1.0_5'inner1 | AATTGGGTGYCARCATAGCAGAATAG |
| (5918 – 5945) | tat1.0_3'outer2 | CGSTGTYTCCGCTTCTT |
|  | tat1.0_5'inner2 | AGCCCTGGAABCATCC |
|  | tat1.0_3'inner2 | GCTTCTTCCTGCCATAGGA |
|  | GKTH02B05Y | FAM-TGYTGCTTTCATTGCCAAGTTTGTTTCA-BHQ1 |
| *gp120* | gp120_2_FO1 | TAATTGTGGAGGRGAATTTTTCTA |
| (7505 – 7532) | gp120_2_RO5 | CTACTTTATAYTTATATAATTCACTTCTCCAATT |
|  | gp120_2_RO1 | TTTATATAATTCACTTCTCCAATTGTC |
|  | gp120_2_1.2 | FAM-CATACATTGCTTTTCCTACTTCCTGCCA-BHQ1 |
| *nef* | nef_1.0_5'_outer1 | AAGAATYAGACAGGGCTTRGAA |
| (8916 – 8943) | nef_1.0_3'_outer2 | TCCAGTCCCCCCTTTTCTTTT |
|  | nef_1.0_5'_inner1 | ATGGGDRGCAARTGGTCAA |
|  | nef_1.0_3'_inner1 | TAAGTCATTGGTCTTAAAGGYAC |
|  | B_PR2_nef_1.4 | FAM-TGGAGCAATCACAAGTAGCAATACAGCA-BHQ1 |
