# Supplementary material for: HIV-1 genetic diversity and demographic characteristics in Bulgaria
Source: PLoS One. 2019 May 28;14(5):e0217063. doi: 10.1371/journal.pone.0217063 (PMC6538145; doi:10.1371/journal.pone.0217063)
Supplement: S2 Table — (DOCX) [file pone.0217063.s003.docx]

**S2 Table. MHA B/non-B validation panel**

| **HIV-1 isolate name** | **Subtype** |
| --- | --- |
| TZ_A341 | A |
| TZ_A173 | A |
| KE_KNH1199 | A |
| KE_MSA4079 | A |
| FR_BXO8 | B |
| US_90US_873 | B |
| TH_NP1538 | B |
| TH_NP1635 | B |
| TH_MU2028 | B |
| TH_MU2008 | B |
| TH_MU2023 | B |
| TZ_A388 | C |
| TZ_BD22_11 | C |
| TZ_CO6721 | C |
| TZ_A301 | C |
| US_98US_MSC5016 | C |
| US_98US_MSC3018 | C |
| IN_93IN905 | C |
| IN_95IN21803 | C |
| UG_99UGA08483 | D |
| TZ_A280 | D |
| UG_99UGD23550 | D |
| UG_99UGK09958 | D |
| UG_98UGJ32228 | D |
| UG_98UGG57140 | D |
| NG_010 | G |
| NG_059 | G |
| TH_NP1650 | CRF01_AE |
| TH_90TH_CM244 | CRF01_AE |
| TH_MO66 | CRF01_AE |
| US_98US_MSC3012 | CRF01_AE |
| US_98US_MSC1120 | CRF01_AE |
| CM_01CM_CAM1475MV | CRF02_AG |
| A0151 | CRF02_AG |
| NG_92NG003 | A1G |
